# Boy in the Barrel: Excruciating Paroxysmal Pain Disorder Associated With an SCN9A Gain‐of‐Function Variant

**DOI:** 10.1111/jns.70142

**Published:** 2026-07-12

**Authors:** Pedro Jose Tomaselli, Rodrigo Siqueira Soares Frezatti, Christopher J. Record, Maria Cristina Lopes Schiavoni, Natalia Dominik, Jana Vandrovcova, Mary M. Reilly, Wilson Marques

**Affiliations:** ^1^ Department of Neurology School of Medicine at Ribeirao Preto, University of Sao Paulo Ribeirao Preto Brazil; ^2^ Department of Neuromuscular Diseases UCL Queen Square Institute of Neurology London UK; ^3^ INCT Translational Medicine Universidade Federal do Rio Grande do Sul Porto Alegre Brazil

**Keywords:** carbamazepine, Nav1.7, paroxysmal pain, SCN9A, small‐fibre neuropathy

## Abstract

**Background and Aims:**

Gain‐of‐function variants in SCN9A, encoding the Nav1.7 sodium channel, cause inherited painful neuropathic disorders. We report a young man with severe childhood‐onset heat‐triggered paroxysmal pain, autonomic dysfunction, skeletal abnormalities, and a de novo SCN9A p.Ile234Thr variant, emphasizing the diagnostic and therapeutic relevance of comprehensive phenotyping.

**Case Report:**

The patient developed excruciating lower‐limb pain in early childhood, partially relieved by prolonged immersion in cold running water. Evaluation demonstrated marked small‐fibre dysfunction, absent sympathetic skin responses, impaired sweating, absent lower‐limb pain‐related evoked potentials, loss of dermal and epidermal nerve fibres, pronounced small myelinated fibre loss on sural nerve biopsy, and mild large‐fibre involvement. Whole‐exome sequencing identified the de novo pathogenic SCN9A variant c.701 T>C; p.Ile234Thr. Carbamazepine led to more than 90% pain improvement and substantial functional recovery.

**Interpretation:**

This case expands the clinical spectrum associated with SCN9A p.Ile234Thr and illustrates how genetic diagnosis may directly guide treatment. The associated large‐fibre abnormalities and acetabular dysplasia are interpreted cautiously, as their relationship to SCN9A dysfunction remains uncertain.

## 1 Background and Aims

Dominant gain‐of‐function variants in SCN9A may cause inherited painful neuropathic disorders, including erythromelalgia, paroxysmal extreme pain disorder, and painful small‐fibre neuropathy. The p.Ile234Thr variant has been associated with severe pain beginning in childhood, but also with phenotypic complexity that may include features suggesting reduced pain perception in some territories [1, 2].

Sodium channel blockers may alleviate pain in selected patients, although treatment response cannot be reliably predicted from the clinical syndrome alone. Functional work has shown that carbamazepine can normalize activation and attenuate thermal hyperexcitability associated with Nav1.7‐Ile234Thr [3].

We report a young Brazilian man with excruciating pain, autonomic dysfunction, acetabular dysplasia, marked small‐fibre involvement, and dramatic response to carbamazepine, with the aim of emphasizing the relevance of integrating clinical, neurophysiological, pathological, and genetic findings in severe painful neuropathic disorders.

## 2 Case Report

**FIGURE 1:**
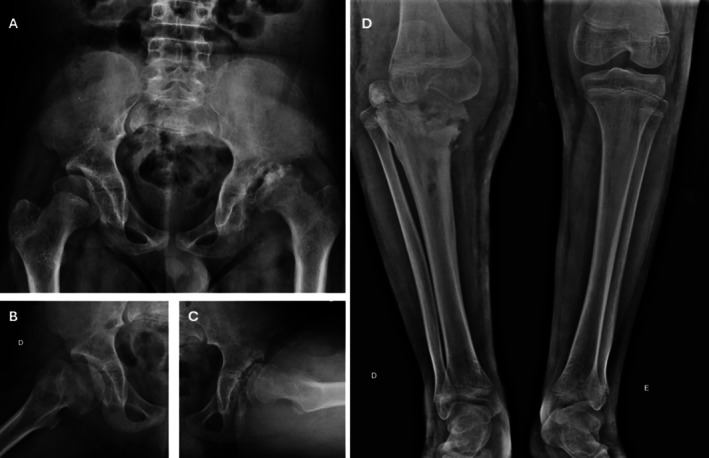
Pelvic and lower‐limb radiographs. The acetabula have a steep and shallow contour. The femoral heads have lost their normal rounded shape, with reduced centre‐edge angle and shallow acetabulum, consistent with acetabular dysplasia, accompanied by deformity and degenerative changes of the femoral heads, mainly on the left side (A–C). The right knee shows degenerative changes, including joint space narrowing, osteophyte formation, and subluxation (D).

A 23‐year‐old male patient, born to healthy and unrelated Brazilian parents, presented with a complex disorder characterized by excruciating pain episodes, heat intolerance, hypohidrosis, and difficulty walking due to bilateral acetabular dysplasia, diagnosed in his first year of life (Figure 1A–D; Video S1). Motor milestones were delayed, and he walked independently at 30 months of age. According to his parents, his gait was laborious and initially attributed to the hip disorder.

In his third year of life, he developed excruciating episodes of lancinating pain and hyperaemia localized distally in the lower limbs, lasting from minutes to hours. Over time, the episodes became more frequent and prolonged and eventually became nearly continuous. Heat was a potent trigger and a major contributor to his deteriorating quality of life, as he lived in a rural area in northeastern Brazil, where temperatures vary throughout the year from 20°C to 30°C (68 to 86 F). Cold‐water baths and creek baths alleviated the pain. His family therefore adapted a barrel to hold him safely in running cold river water, where he spent much of his time submerged to alleviate suffering (Figure S1). Sleeping was challenging and often required the use of wet sheets to bring some comfort. He denied rectal pain or exacerbations with defecation, and denied mandibular pain with eating or yawning.

Neurological examination at 14 years of age revealed normal cognition. Cranial nerve examination, including eye movements, tongue, and palate, was normal. There was mild hip flexion weakness attributed to his acetabular problem. Deep tendon reflexes were normal throughout, and plantar responses were flexor. There was hyperalgesia to pinprick in the lower limbs. Vibration sense, proprioception, and coordination were normal. Mild postural tremor was present in both upper limbs. There was no muscle atrophy or fasciculations. The skin on his lower limbs exhibited pronounced hyperkeratosis and significant oedema, likely related to prolonged water exposure. Brain MRI was normal.

Nerve conduction studies were performed at 20 years of age. The right ulnar motor response was normal, with a compound muscle action potential amplitude of 12.9 mV (reference value > 5.5 mV) and conduction velocity of 66.7 m/s (reference value ≥ 49 m/s). The right radial sensory nerve action potential amplitude was 24.8 microV (reference value > 15 microV), with conduction velocity of 47.5 m/s (reference value > 48 m/s). In the lower limb, the right sural sensory nerve action potential amplitude was reduced at 2.1 microV (reference value ≥ 6 microV), and the right peroneal compound muscle action potential amplitude was reduced at 0.9 mV (reference value ≥ 2.9 mV). Needle electromyography was limited by patient tolerance and was performed only in the biceps, anterior tibialis, and first dorsal interosseous muscles, showing normal motor unit action potentials.

**FIGURE 2:**
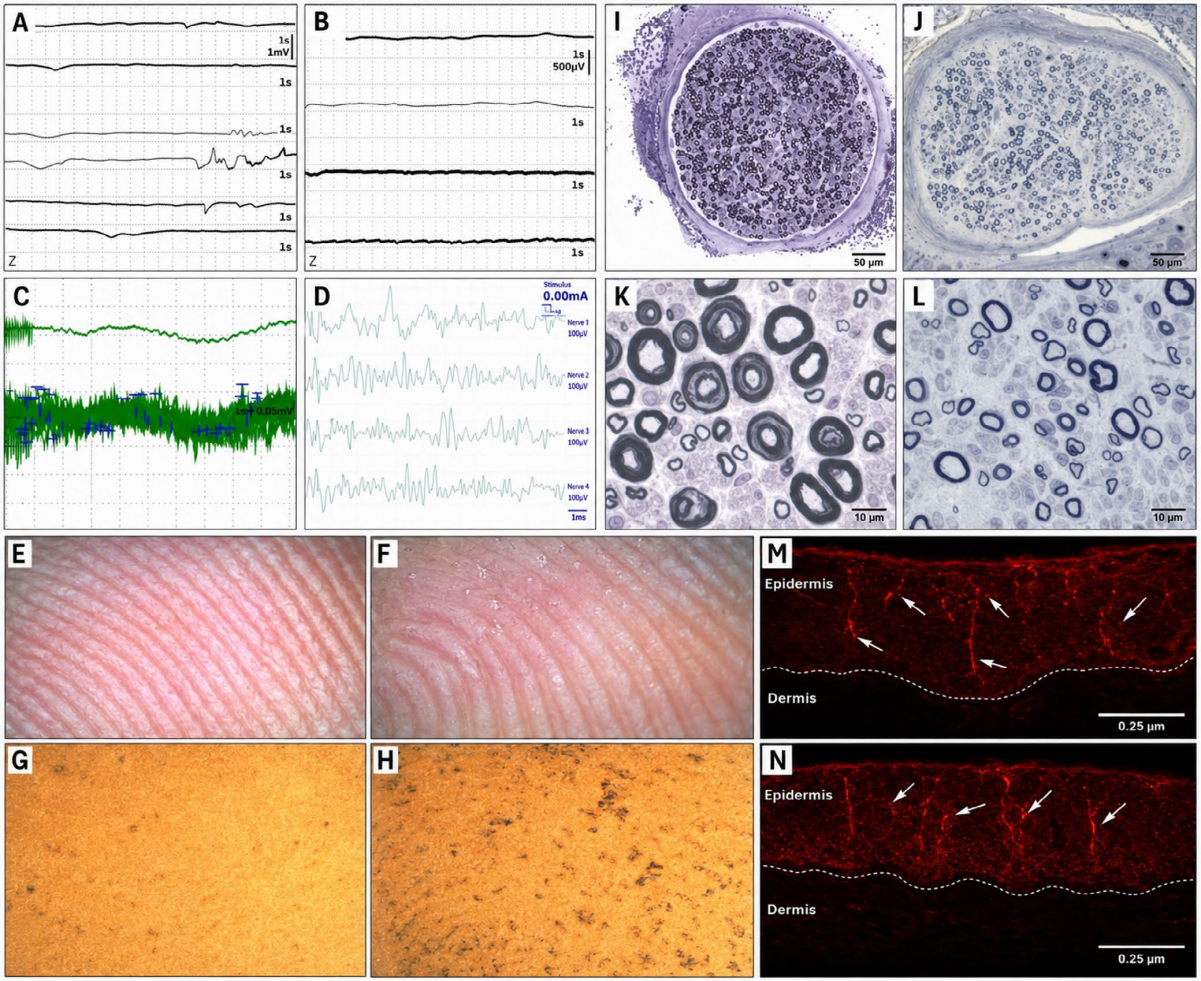
Neurophysiological tests, small‐fibre assessment, and nerve and skin biopsy. Sympathetic skin response to sound and electrical stimuli revealed no response in the upper and lower limbs (A and B). Pain‐related evoked potentials were normal in the upper limbs and absent in the lower limbs (C). Sural nerve cutaneous silent period was absent (D). Sweating analysis in the index finger showed marked impairment (E) compared with a normal control (F). The activity and density of active sweat glands on the volar surface of the left ring finger were assessed using a USB digital microscope (Optic Zoom 1000×, 2.0MP) for real‐time images. Quantification revealed markedly reduced density (G) compared with normal controls (H). The analysis was conducted using conventional quantitative filter paper (2 × 2 cm) soaked with a hydrochromic solution (acetone and bromophenol blue), where the dark dots indicate active sweat gland activity. Semi‐thin sections stained with toluidine blue from a sural nerve biopsy revealed pronounced loss of small myelinated fibres (J–L) and the presence of some large myelinated fibres with disproportionately thin myelin sheaths relative to their axons, compared with the control (I–K). No dermal or epidermal nerve fibres were observed on PGP9.5 immunostaining with CY3 fluorescence (M), in contrast to the normal control (N).

Small‐fibre and autonomic evaluation demonstrated absent sympathetic skin responses in the upper and lower limbs, normal pain‐related evoked potentials in the upper limbs and absent responses in the lower limbs, absent sural nerve cutaneous silent period, and markedly impaired sweating (Figure 2A–H). Sural nerve biopsy revealed pronounced loss of small myelinated fibres and mild but consistent involvement of larger fibres (Figure 2I–L). Skin biopsy showed absence of dermal and epidermal nerve fibres by PGP9.5 immunostaining (Figure 2M,N).

An extensive laboratory workup for acquired causes, including metabolic, vitamin deficiency, and inflammatory conditions, was unremarkable. Whole‐exome sequencing was performed after recruitment through the International Consortium for Genetic Neuromuscular Disorders (ICGNMD; study ID IC_BAP_00942). WES revealed a heterozygous pathogenic SCN9A missense variant, NM_001365536.1:c.701 T>C; p.Ile234Thr, located at a highly conserved amino acid in the N‐terminus of the linker between S4 and S5 in domain I (D1/S4‐S5) of Nav1.7. The variant corresponds to dbSNP rs1698638581 and ClinVar Variation ID 915877. Parental testing confirmed that the variant had arisen de novo. No relevant variants were identified in genes associated with neuropathy or acetabular dysplasia.

Patient had been treated with amitriptyline, duloxetine and gabapentin although without response. Following genetic result, carbamazepine was introduced and progressively titrated to 200 mg three times daily, resulting in more than 90% pain improvement. This allowed the patient to stop using the water barrel for pain relief and to initiate normal daily activities, including attending school. The same SCN9A variant has previously been shown to produce marked functional effects on Nav1.7 activation and to be responsive to carbamazepine in experimental models.1–3.

## 3 Interpretation

This case illustrates a severe and complex SCN9A‐related painful neuropathic disorder associated with the de novo p.Ile234Thr variant. The clinical picture included early‐onset heat‐triggered distal lower‐limb pain, autonomic dysfunction, marked small‐fibre impairment, and a striking response to carbamazepine. Although the phenotype overlapped with paroxysmal pain disorders, the absence of typical rectal or mandibular triggers and the prominent small‐fibre and autonomic involvement support phenotypic complexity rather than a single classic syndrome [1, 2, 3].

Huang et al. demonstrated that the p.Ile234Thr variant may produce a dual functional effect. Although the variant causes a major hyperpolarizing shift in activation consistent with gain of function, expression in dorsal root ganglion neurons may also lead to marked depolarization and loss of excitability in a subset of neurons, providing a mechanism for coexistence of severe pain and reduced pain perception [2]. Our patient shared the severe pain phenotype and marked small‐fibre involvement, but we did not document painless fractures, corneal anesthesia, or a clear clinical syndrome of generalized pain insensitivity. The absent lower‐limb pain‐related evoked potentials and loss of epidermal nerve fibres may, however, be compatible with severe structural and functional small‐fibre impairment, and should be interpreted in the context of this previously described dual phenotype.

The acetabular dysplasia is intriguing but remains speculative as part of the SCN9A phenotype. Recessive loss‐of‐function SCN9A variants are associated with congenital insensitivity to pain and skeletal complications, often secondary to unperceived injury. Conversely, abnormal limb development has also been reported in association with gain‐of‐function SCN9A‐related small‐fibre neuropathy [4, 5]. Because no additional genetic cause of acetabular dysplasia was identified in this patient, a relationship with SCN9A dysfunction is possible; however, this cannot be established from a single case.

The large‐fibre abnormalities also require cautious interpretation. Nav1.7 is predominantly expressed in nociceptive and autonomic small fibres and would not typically be expected to directly cause a large‐fibre neuropathy. Reduced sural and peroneal amplitudes may have been influenced by technical factors, including marked lower‐limb oedema and skin hyperkeratosis presumably due to recurrent water exposure, which can attenuate surface‐recorded responses. Nevertheless, the sural nerve biopsy showed mild large‐fibre involvement, indicating that the electrophysiological abnormalities cannot be dismissed as purely technical. An independent or comorbid process cannot be excluded, and longitudinal follow‐up will be important to clarify whether these findings progress or remain stable.

The dramatic response to carbamazepine supports the concept that genetic diagnosis may have immediate therapeutic consequences in selected monogenic pain disorders. In this case, the variant‐level functional data were directly relevant to treatment choice, consistent with a reverse pharmacogenomics approach in which genotype and channel behavior guide targeted therapy [3].

## Funding

This work was supported by a Medical Research Council (MRC) Strategic Award (MR/S005021/1) to establish the International Centre for Genomic Medicine in Neuromuscular Diseases (ICGNMD). WM Jr. is supported by the Instituto Nacional de Ciência e Tecnologia em Saúde Mental Digital, CNPq/SECTICS/CAPES/FAPs Call No. 46/2024, grant number 409148/2024–5, and by CNPq Research Productivity Call PQ 2021, grant number 310378/2021.

## Consent

Written informed consent was obtained from the patient for publication of this case report, including the accompanying images and videos provided as Supporting Information.

## Supporting information


**Figure S1:** Patient immersed in a water‐filled barrel with water flowing overhead. It illustrates the adaptive strategy used for pain relief.

**Video S1:** Video illustrating the patient walking with waddling gait, highlighting the severity of hip dysplasia.

## Data Availability

The data that support the findings of this study are available on request from the corresponding author. The data are not publicly available due to privacy or ethical restrictions.
