# Supplementary figures and images for: Boy in the Barrel: Excruciating Paroxysmal Pain Disorder Associated With an SCN9A Gain‐of‐Function Variant
Source: J Peripher Nerv Syst. 2026 Jul 12;31(3):e70142. doi: 10.1111/jns.70142 (PMC13357776; doi:10.1111/jns.70142)

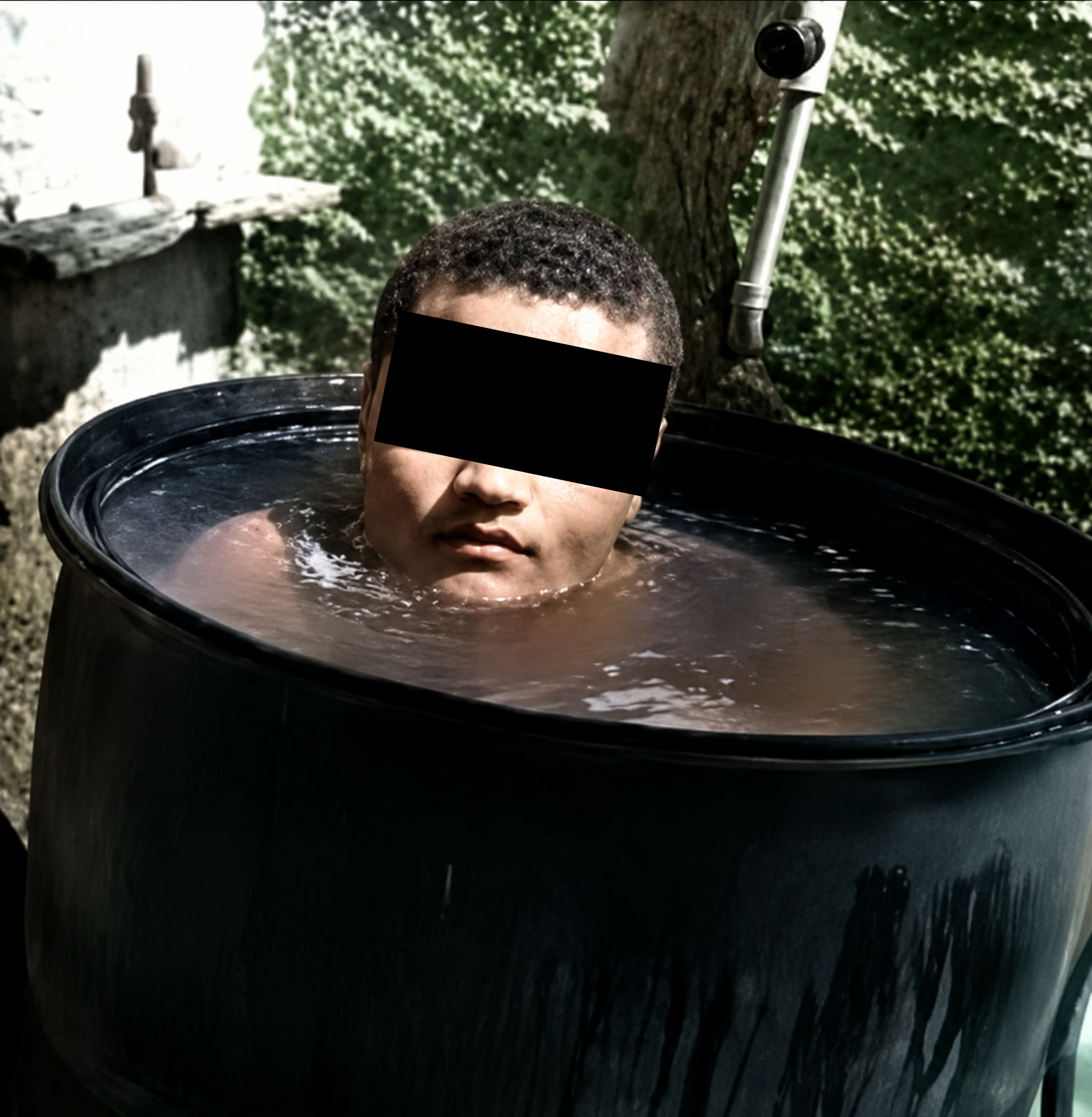

Supplement: Supplementary file 1 — Figure S1: Patient immersed in a water‐filled barrel with water flowing overhead. It illustrates the adaptive strategy used for pain relief. [file JNS-31-0-s002.tiff]
